# Spatial and compositional variation in the fungal communities of organic and conventionally grown apple fruit at the consumer point-of-purchase

**DOI:** 10.1038/hortres.2016.47

**Published:** 2016-10-05

**Authors:** Ahmed Abdelfattah, Michael Wisniewski, Samir Droby, Leonardo Schena

**Affiliations:** 1 Dipartimento di Agraria, Università Mediterranea di Reggio Calabria, Località Feo di Vito, 89124 Reggio, Calabria, Italy; 2 U.S. Department of Agriculture-Agricultural Research Service (USDA-ARS), 2217 Wiltshire Road, Kearneysville, WV 25430, USA; 3 ARO, Department of Postharvest and Food Sciences, The Volcani Center, 68 HaMccabim Road, Rishon LeZion 7505101, Israel

## Abstract

The fungal diversity in harvested apples from organic or conventional management practices was analyzed in different fruit locations (stem end, calyx end, peel, and wounded flesh) shortly after fruit purchase (T1) and after 2 weeks of storage (T5). A total of 5,760,162 high-quality fungal sequences were recovered and assigned to 8,504 Operational Taxonomic Units. Members of the phylum *Ascomycota* were dominant in all samples and accounted for 91.6% of the total number of detected sequences. This was followed by *Basidiomycota* (8%), *Chytridiomycota* (0.1%), and unidentified fungi (0.3%). Alpha and beta diversity analyses revealed the presence of significantly different fungal populations in the investigated fruit parts. Among detected fungi, the genus *Penicillium* prevailed in the peel and in the wounded flesh while *Alternaria* spp. prevailed in the calyx and stem end samples that included apple core tissues. Several taxonomic units that appear to be closely related to pathogenic fungi associated with secondary human infections were present in peel and wounds. Moreover, significantly different populations were revealed in organic and conventional apples and this result was consistent in all investigated fruit parts (calyx end, peel, stem end, and wounded flesh). Several unique taxa were exclusively detected in organic apples suggesting that management practices may have been a contributing factor in determining the taxa present. In contrast, little differences were revealed in the two assessment times (T1 and T5). Results of the present study represent an advancement of the current knowledge on the fungal microbiota in collected fruit tissues of apple.

## Introduction

Fruits serve as hosts to many microorganisms that colonize the surface (epiphytes) or live within (endophytes) their tissues. They represent a primary habitat and source of energy in the lifecycle of many fungi, some of which are host-specific and whose survival depends on the presence of their host. Several of the fungi resident on an in fruit are known to be phytopathogenic and are responsible for significant economic losses, before and after harvest, while other resident microorganisms are considered beneficial and can influence the severity of disease symptoms by directly interacting with pathogens or by inducing resistance in the host.^1,2^ For example, fungal endophytes have been shown to decrease (pathogen antagonism) or increase (pathogen facilitation) plant disease severity.^3^

Conventional disease management strategies have relied heavily on the use of synthetic chemical compounds to control these pathogenic fungi; however, an ever increasing awareness of the environmental and health risks associated with the use of synthetic, chemical fungicides continues to drive research aimed at developing safer and eco-friendlier alternative control practices.^4^ Thus, organic production strategies for the production of crops has greatly increased in the past 20 years and continues to grow. The United States Department of Agriculture, defines organic production as an ecological production system that integrates cultural, biological and mechanical practices that foster resource cycling, ecological balance and biodiversity. This approach differs from conventional management systems in its use of a variety of alternative disease management strategies rather than the use of synthetic, chemical pesticides.^5^ In this context, there is growing recognition of the valuable role that microorganisms have in plant health, nutrient acquisition, and even stress tolerance.^6,7^

The domesticated apple (*Malus×domestica* Borkh, family Rosaceae, tribe Pyreae) is a major temperate fruit crop, and is grown in large numbers worldwide. Apples are grown using both organic (sustainable) and non-organic (conventional) management practices. Like many other fruit crops, apple is subject to infections by several different phytopathogens and is colonized by a number of different microorganisms. Current knowledge about the apple microbiota is largely focused on species of microbes that cause disease and thus pose economic threats, or in some cases, natural antagonists that could be used as biological control agents against these pathogens.^8,9^ Differences in orchard production strategies, yield, and fruit quality between organic and conventional management systems have been documented, including differences in microbial diversity.^10,11^ The microbial studies, however, have been mainly based on the isolation and culturing of microorganisms from the phyllosphere and rhizosphere of trees,^10,12^ or sometimes have focused only on the population dynamics of a single pathogen.^11^ Thus our current understanding of the fruit microbiota is largely and exclusively based on the ability to isolate, culture, and identify the most abundant microorganisms which are estimated to represent only a small fraction of the total estimated microbial diversity.^13^

As a consequence, little is known about the fungal microbiota of apple or other fruits. In particular, little information is available about the complex interactions that occur between microbial populations, host tissues and pathogens, or how they are affected by biotic and abiotic elicitors of defense mechanisms, or by the environment and different cropping systems. In this context, recent advances in -omics technologies (genomics, transcriptomics, metabolomics, proteomics, and metagenomics) can play a crucial role in increasing our understanding of fungal biology in relation to food crops, and the effects of alternative control methods, such as biological control. In particular, massive sequencing of PCR amplicons of specific barcode genes (amplicon metagenomics or metabarcoding) has proved to be a powerful culture-independent technique for investigating microbial diversity and for determining the relative quantity of community members in environmental samples.^14,15^ The identification and quantification of endophytic and epiphytic microflora present in and on plants provide an opportunity to investigate communities of microbes that exist on plant surfaces, how they interact, and how they change over time. This knowledge will provide the foundation for a systems approach to disease control and will lead to the ability to create synthetic communities of organisms for enhanced disease management.^1^

In the present study, a metagenomics approach, based on the fungal ITS2 region, was utilized to assess the fungal diversity of organic and conventional ’Red Delicious’ apple fruit. The objective of the study was to investigate: (i) the composition of fungal communities in different fruit parts, including stem end, calyx end, peel, and wounded flesh (‘location’ effect); (ii) changes in the composition of fungal communities in the above fruit parts over a two-week period of storage at room temperature (‘time’ effect); and (iii) the potential impact of management practices (organic versus conventional) on apple fungal communities (‘practices’ effect).

## Materials and methods

### Experimental design

Experiments were conducted on collected ‘Red Delicious’ fruit, purchased at a local supermarket (Ranson, WV, USA) that was grown using conventional management practices or labeled organic. All the fruit was derived from orchards in the state of Washington, USA. The organic fruit were labeled as, ‘Washington Extra Fancy Red Delicious,’ certified organic by the Washington State Department of Agriculture, originating from the Rainer Fruit Company, Selah, WA, USA. The non-organic or ‘conventional’ fruit were also labeled ‘Washington Extra Fancy Red Delicious,’ originating from the Rainer Fruit Company, Selah, WA, USA. The local distributor for both fruit was Foodhold USA, Landover, MD, USA. Fruits were purchased on 9 April 2015, which also means that the apples had been collected in the fall of 2014, and placed in controlled atmosphere storage until they were purchased by the distributor. While the ethylene inhibitor, 1-MCP is commonly used to preserve the quality of non-organic apples in storage, its use on organically grown apples is not allowed. All samples were processed and sampled within a day after purchasing (T1) and after two weeks (T5) of storage at room temperature (~20 °C and 60% RH). All fruits were initially injured at ten equidistant points in the equatorial zone with a small nail (approx. 0.5 mm wide and 3 mm deep) in order to have wounds to sample. Three different fruit locations (calyx end, stem end, and equatorial zone) were sampled in both the conventionally and organically grown apples at both assessment times. The equatorial zone was further subdivided into peel and wounded flesh (mesocarp) samples. Sterilized gloves were used during the handling of all fruit treatments and sampling.

Wounded flesh (WF) and surrounding mesocarp tissues were sampled using a cork borer (diameter 3 mm) and peel tissues were removed with a sterile razor prior to saving the sample. Apple peel (PE) samples were collected from non-wounded parts of the same fruits using a fruit peeler. Calyx end (CE) and stem end (SE) samples were also collected from the same fruits by excising the core of the apples with a cork borer (diameter 10 mm). The core was then divided in three equal parts, SE, middle (seed area) and CE. The middle part of the core was discarded and the SE and CE were collected and analyzed separately. A total of 15 fruits from the conventionally and organically grown apples were sampled at both assessment times (T1 and T5). Samples were randomly mixed to obtain either 5 (CE and SE) or 3 (WF and PE) biological replicates. Collected samples were immediately frozen in liquid nitrogen and stored at −80 °C prior to lyophilization.

### DNA extraction, amplification and sequencing

Lyophilized samples were homogenized in a 2010 Geno/Grinder (SPEX SamplePrep, Metuchen, NJ, USA) using autoclaved metal beads. Total DNA was extracted using the Wizard Genomic DNA Purification Kit (Promega, Madison, WI, USA) according to the manufacturer’s protocol. DNA samples were analyzed using a Nanodrop spectrophotometer (Thermo Fisher Scientific, Inc., Waltham, MA, USA) and the total DNA concentration was adjusted to 50 ng/μl^−1^. The fungal ITS2 region was amplified using the universal primers ITS3_KYO2 and ITS4 to amplify the ITS2 region of ribosomal DNA.^16^ Both primers were modified to include Illumina adapters (www.illumina.com) for subsequent multiplexing. PCR reactions were conducted in a total volume of 25 μl containing 12.5 μl of KAPA HiFi HotStart ReadyMix (Kapa Biosystems, Wilmington, MA, USA), 1.5 μl of each primer (10 μm), and 1 μl of DNA template. Reactions were incubated in a T100 thermal cycler (Bio-Rad, Hercules, CA, USA) for 3 min at 98 °C followed by 30 cycles of 30 s at 95 °C, 30 s at 50 °C and 30 s at 72 °C. All reaction cycles ended with a final extension of 1 min at 72 °C. Nuclease-free water (Qiagen, Valencia, CA, USA) replaced template DNA in negative controls. All amplicons and amplification mixtures including negative controls were sent to the DNA Services Facility (University of Illinois, Chicago, IL, USA) for sequencing using Illumina MiSeq V3 (2×300 bp) chemistry.

### Data analysis

Paired-end reads were merged using PEAR 0.9.6 Paired-End reAd merger with default parameters.^17^ The CLC genomics workbench V8 (Qiagen) was used for primer and quality trimming with a minimum of Q20. Sequences without either primer were discarded. Chimeric sequences were identified and filtered using VSEARCH 1.4.0.^18^ The UCLUST algorithm^19^ of the software package QIIME 1.9.1^20^ was used to cluster sequences at a similarity threshold of 97% against the UNITE dynamic database released on 31 January 2016.^21^ Sequences that failed to cluster against the database were *de novo* clustered using the same algorithm. The most abundant sequences in each Operational Taxonomic Unit (OTU) were selected as representative sequences and used for the taxonomic assignment using the BLAST algorithm^22^ as implemented in QIIME 1.9.1 (www.qiime.org).

The OTU table was normalized by rarefaction to an even sequencing depth in order to remove sample heterogeneity. The rarefied OTU table was used to calculate alpha diversity indices including Observed Species (Sobs), Chao1, and Shannon metrics. MetagenomeSeq’s cumulative sum scaling was used as a normalization method for other downstream analyses, including taxa relative abundance, β-diversity, and group significance.^23^ Alpha diversities were compared based on a two-sample *t*-test using nonparametric (Monte Carlo) methods and 999 Monte Carlo permutations. Results were visualized in boxplots figures.

The cumulative sum scaling normalized OTU table was analyzed using the Bray Curtis metrics^24^ and utilized to evaluate the β-diversity and construct PCoA plots.^25^ Differential OTU abundance of the most abundant taxa (⩾0.1%) between sample groups were determined using a *t*-test and the Kruskal–Wallis test.^26^ In all tests, significance was determined using 999 Monte Carlo permutations, and the false discovery rate (FDR) was used to adjust the calculated *P*-values and when the FDR *P*<0.05 it was considered significant. Cytoscape 3.3.0 (www.cytoscape.org) was used to analyze the most abundant taxa (⩾0.1%) and construct network figures visualizing the interactions between significantly different taxa (*P*<0.01).

### Identification of representative taxa

Standard QIIME analyses only provide a reliable identification of fungi down to the genus level. Therefore, the identity of most sequences were manually re-checked using BLAST searches of GenBank and Fungal Barcoding Databases (http://www.fungalbarcoding.org/). Furthermore, sequences representative of relevant OTUs were phylogenetically analyzed along with closely related reference sequences to enable their identification with the highest level of accuracy possible.^15,27^

## Results

### Sequencing results

**Figure 1:**
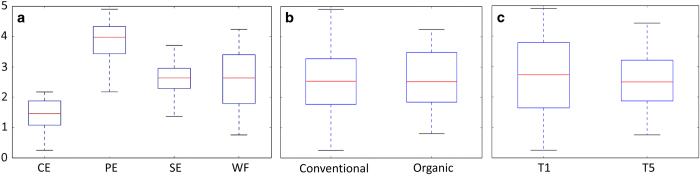
Boxplots visualizing results of the nonparametric two-sample *t*-test based on Shannon diversity to compare the alpha diversity of fungal communities associated to different fruit parts (a), conventional and organic apples (b), and different assessment times (c). Analyzed fruit parts comprised calyx end (CE), peel (PE), stem end (SE), and wounded flesh (WF), and were collected from organic and conventional ‘Golden Delicious’ apples within a day after purchasing (T1), and after two weeks (T5) of storage at room temperature.

A total of 1,835,926 reads were recovered and assigned to 1,591 fungal OTUs after paired-end alignments, quality filtering, and deletion of chimeric, singletons, and plant sequences. The number of sequences in the collected samples varied between 103,720 and 401,759 sequences after collapsing biological replicates, and the number of OTUs varied between 78 and 441 OTUs (Table 1). The analysis of a rarefied OTU table to an even depth of 700 reads per sample revealed a higher number of OTUs in the PE followed by WF, SE samples, based on alpha diversity metrics. CE samples contained the lowest number of OTUs at both assessment times (T1 and T5) and in both conventional and organic apples (Table 1, Figure 1). Both management practice (conventional versus organic) and assessment time (T1 versus T5) had no significant effect on alpha diversity metrics (Table 1; Figure 1).

### Apple fungal communities

**Figure 2:**
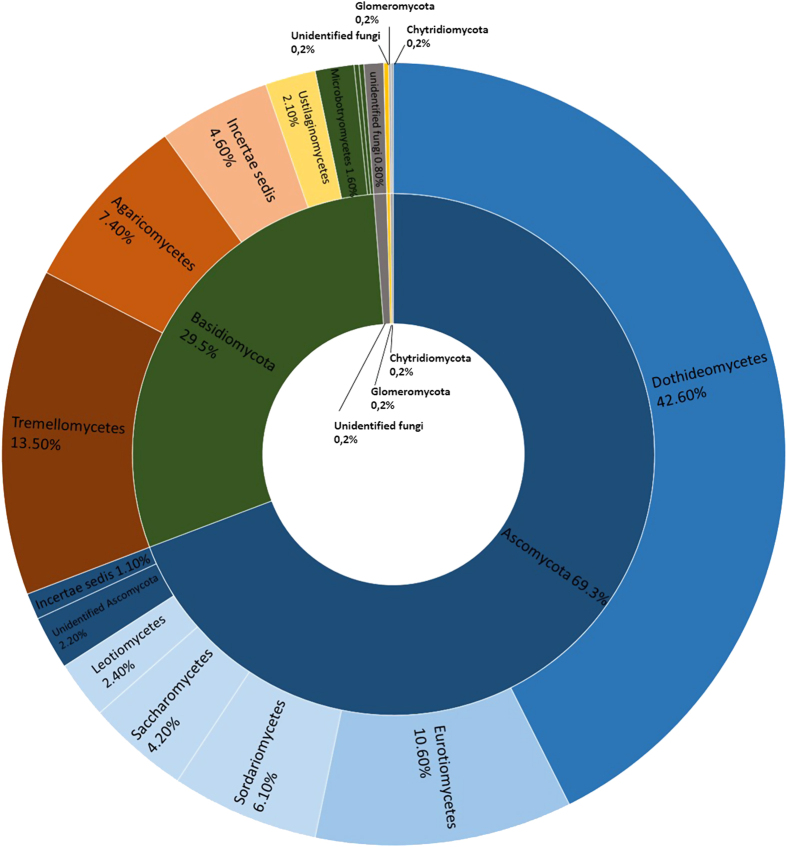
Sunburst chart showing the total relative abundance of fungal phyla (interior circle) and classes (exterior circle) overall detected in investigated samples.

**Figure 3:**
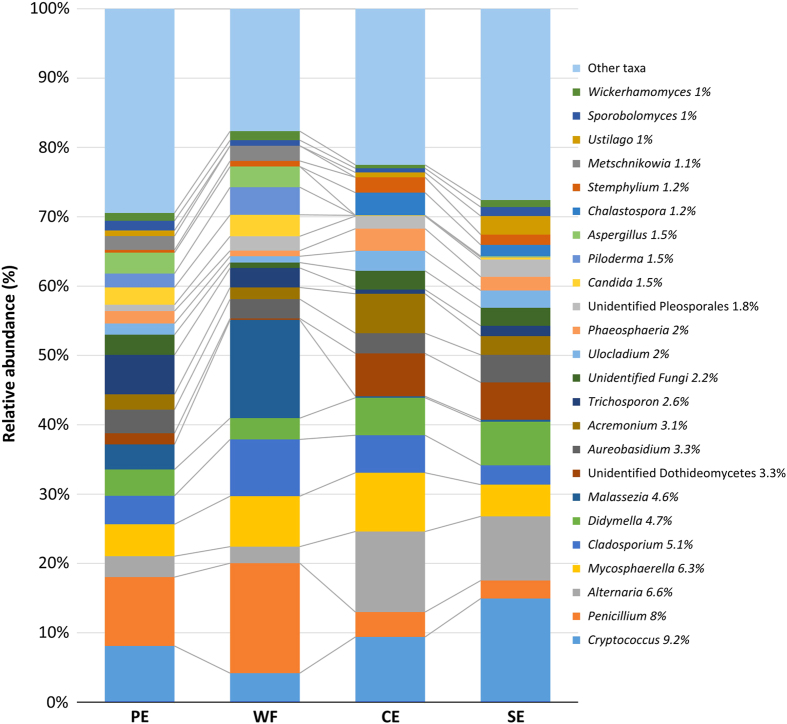
Relative abundance (RA) of fungal genera (RA⩾1%) detected in peel (PE), wounded flesh (WF), calyx end (CE), and stem send (SE) of conventional and organic apples. Fungal genera with a cumulative RA<1% are reported as ‘other taxa’. Percentages reported in the legend along with fungal genera represents the average RA in the four investigated locations.

Members of the *Ascomycota* were the dominant phylum across all samples, accounting for 69.3% of the total number of detected sequences (Figure 2). This was followed by the *Basidiomycota* (29.5%) and unidentified fungi (0.8%). *Glomeromycota* and *Chytridiomycota* were also detected but at a very-low frequency (0.2%) (Figure 2). OTUs within the *Ascomycota* were largely identified as members of the classes *Dothideomycetes* and *Eurotiomycetes* (42.6% and 10.6%, respectively), followed by the classes, *Sordariomycetes* (6.1%), *Saccharomycetes* (4.2%), and *Leotiomycetes* (2.4%). *Basidiomycota* were represented mainly by members of the classes *Tremellomycetes* (13.5%) and *Ustilaginomycetes* (2.1%; Figure 2). Overall, the genera *Cryptococcus* (9.20%), *Penicillium* (8.00%), *Alternaria* (6.60%), *Mycosphaerella* (6.30%), *Cladosporium* (5.10%), *Didymella* (4.70%), and *Malassezia* (4.60%) were the most abundant fungi detected (Figure 3).

BLAST and phylogenetic analyses confirmed the identification of almost all of the taxonomic assignments at the genus level. These analyses also provided the identification of a significant number of taxa down to the level of species. In both the BLAST and phylogenetic analyses great precaution was taken in the identification process. Obtained sequences were considered identified at the level of species only when the detected STs (Sequence Types) clustered with a single reference species and when they were clearly differentiated from all other currently known related species. This approach was easier to apply with genera such as *Trichosporon*, *Malassezia*, and *Acremonium* (Supplementary Material), while the identification of species within genera such as *Penicillium*, *Didymella*, and *Alternaria* was much more problematic due to the luck of reliable reference sequences and/or the high similarity or even identity of ITS2 with related species (data not shown).

### Compositional differences in the fungal microbiota of organic and conventionally grown apples

**Figure 4:**
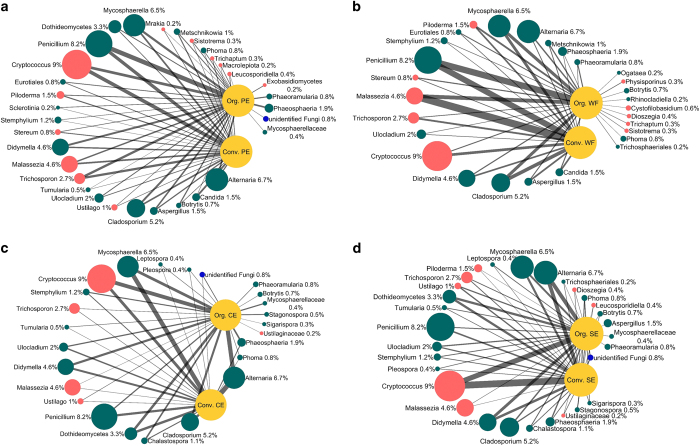
Network figures comparing fungal populations of conventional (Conv.) and organic (Org.) apples in the calyx end (**a**), stem end (**b**), wounded flesh (**c**), and peel (**d**). Networks were constructed regardless of time (using data from both T1 and T5 assessment times) and considering only significantly different taxa (*P*<0.01) with a cumulative RA in organic and conventional apples ⩾0.1%. Investigated samples were represented by large yellow nodes. Detected taxa were represented by green (Ascomycota), red (Basidiomycota), and blue (Unidentified fungi) nodes. The size of nodes is proportional to the cumulative RA of each taxon in all investigated samples. Similarly, the percentage values reported along taxa represent the cumulative RA of each taxon in all investigated samples. On the contrary, the width of line connecting samples and fungal taxa is proportional to the amount of each specific taxon in each specific sample.

**Figure 5:**
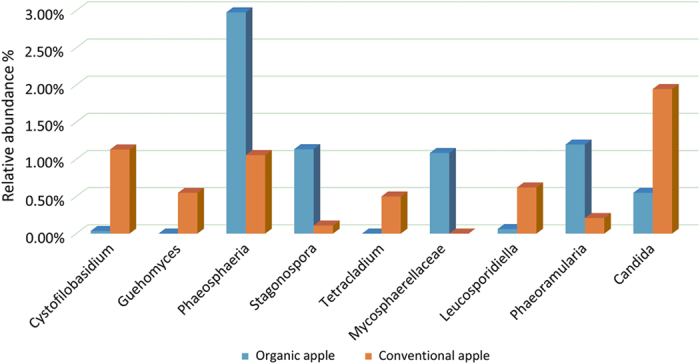
Representative, fungal taxa detected with a significantly different relative abundance in organic and conventional apples.

Alpha diversity metrics did not reveal significant differences between organic and conventionally grown apples, thus indicating a similar level of microbial diversity (Figure 1). Several unique taxa, however, were exclusively detected in organic apples (Figure 4). More specifically, 3, 5, 6 and 10 taxa were exclusively detected in SE, PE, CE, and WF samples, respectively, obtained from organic apples (Figure 4). Additional differences between organic and conventionally grown apples were related to differences in the relative abundance (RA) of detected taxa (Figure 4; Figure 5). Interestingly, among the taxa detected with a significantly different RA, *Ascomycota* were more prevalent in samples obtained from organic apples, while *Basidiomycota* were more abundant in samples from conventionally grown apples. For example, considering the cumulative taxa detected in the four sampled locations (CE, SE, WF, and PE), the Ascomycota *Phaeoramularia*, *Stagonospora, Phaeosphaeria,* and Unidentified Mycosphaerellaceae had a higher RA in organic apples, while the Basidiomycota *Cystofilobasidium*, *Guehomyces*, and *Leucosporidiella* were more abundant in conventionally grown apples (Figure 5). These differences in abundance were more evident for the low abundant taxa, while the RA of the most abundant genera were not significantly impacted by management practice (organic versus conventional) (Figure 4).

In agreement with the findings stated above, the analysis of beta diversity, regardless of sampling time, revealed significant differences between organic and conventionally grown apples in all of the different specific locations (CE, PE, SE, and WF) of the apple that were sampled and analyzed (Table 2).

### Spatial differences in the composition of microbial communities present on harvested apples

**Figure 6:**
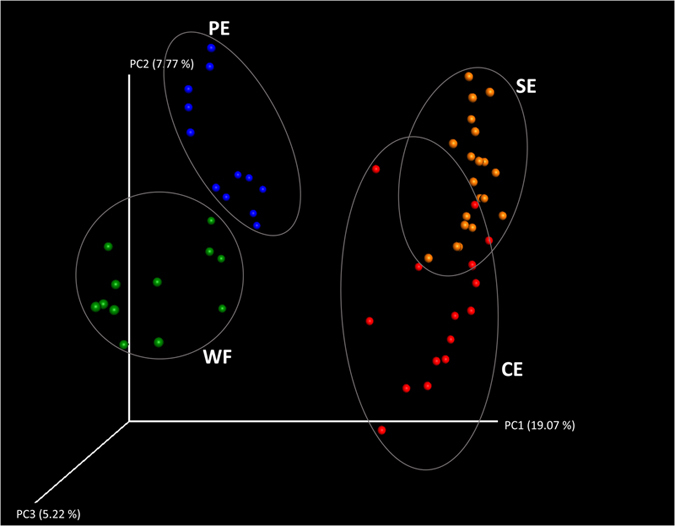
Principal coordinate analysis (PCoA) of fungal populations associated to apples calyx end (CE), stem end (CE), Peel (PE), and Wounded flesh (WF) based on the beta diversity metric Bray Curtis. Analyses were performed considering sequences from both assessment times (T1 and T5) and from both conventional and organic apples.

The Shannon index, a measure of alpha diversity, revealed significant differences between the different portions of the apple fruit that were sampled, except between WF and SE (Table 3; Figure 1). The existence of significant differences was confirmed by the analysis of beta diversity using the nonparametric test, Permanova (Table 2). Results of the statistical analysis indicated that the four locations (CE, SE, WF, and PE) on the fruit possessed significantly different fungal communities in both conventional and organic apples, regardless of the assessment time (T1 versus T5; Table 2). Furthermore, the plot of the Principal Coordinate Analysis (PCoA) collectively analyzing both organic and conventionally grown apples clearly demonstrated a clear clustering based on location (Figure 6). In addition, the presence of two larger clusters separating CE and SE samples from PE and WF samples were also identified, revealing a higher similarity between SE and CE samples than between PE and WF samples (Figure 6).

**Figure 7:**
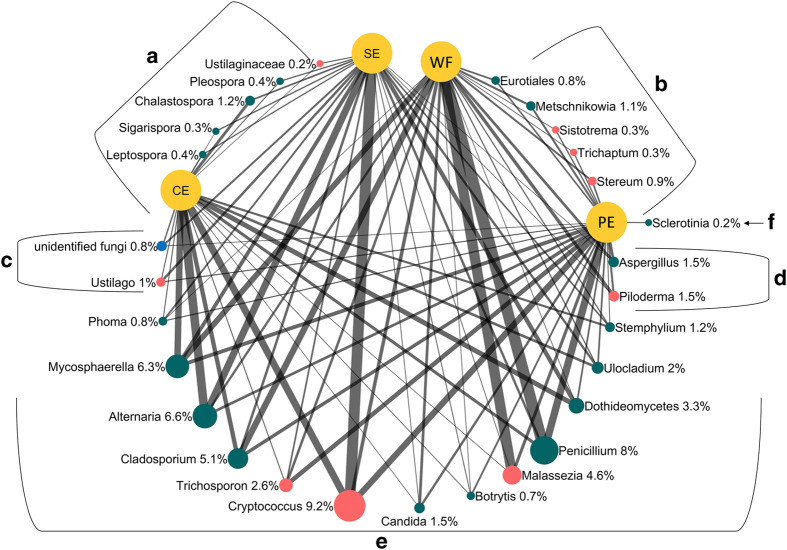
Network figure comparing fungal populations detected in the calyx end (CE), stem end (CE), wounded flesh (WF), and peel (PE) of apple fruit. Networks were constructed using significantly different taxa (*P*<0.01) with a cumulative RA⩾0.1% and sequences from both assessment times (T1 and T5), and from both conventional and organic apples. Investigated samples were represented by large yellow nodes. Detected taxa were represented by green (Ascomycota), red (Basidiomycota), and blue (Unidentified fungi) nodes. The size of nodes is proportional to the cumulative RA of each taxon in all investigated samples. Similarly, the percentage values reported along taxa represent the cumulative RA of each taxon in all investigated samples. On the contrary, the width of line connecting samples and fungal taxa is proportional to the amount of each specific taxon in each specific sample location. Identified groups comprised taxa shared by all investigated locations (**e**), and taxa shared by CE and SE (**a**), WF and PE (**b**), CE, SE, and PE (**c**), and SE, PE, and WF (**d**), and unique to PE (**f**).

The fungal community shared among different fruit parts can be divided into six groups when only significantly different taxa (*P*⩽0.01) and the most abundant taxa (⩾0.1%) are considered (Figure 7). Although the most abundant group representing about the 50% of the total RA (E), comprised fungal taxa shared by all four of the sampled locations, several fungal taxa were only present in CE and SE samples (group A), WF and PE samples (group B), CE, SE, and PE samples (group C) and SE, PE, and WF samples (group D). The last group (F) was represented by a single taxa (*Sclerotinia*) that was exclusively present in PE samples (Figure 7).

The thickness of the lines connecting fungal taxa and samples reflects the RA of fungal taxa in the different sampled locations and illustrates how the RA contributed to differentiating the fungal communities at the different sites (Figure 7). For instance, *Penicillium* was much more abundant in WF samples than in PE samples and much less abundant in CE and SE samples. In contrast, *Alternaria* was more abundant in CE and SE samples and less abundant in PE and WF samples. *Malassezia* was more abundant in WF samples than it was in other fruit locations. Members of the *Dothideomycetes* were more abundant in CE samples (61.1%) than they were in PE samples (28.6%) (data not shown). *Eurotiomycetes* were more abundant in WF samples (20.6%) and PE samples (15.4%) than they were in CE (3.8%) and SE (2.7%) samples. The RA of the *Saccharomycetes* was similar in PE and WF (7.4 and 7.3%) samples, which both had a greater RA than CE and SE (0.8% and 1.4%, respectively) samples (data not shown).

### Effect of storage time on the composition of apple fungal communities

**Figure 8:**
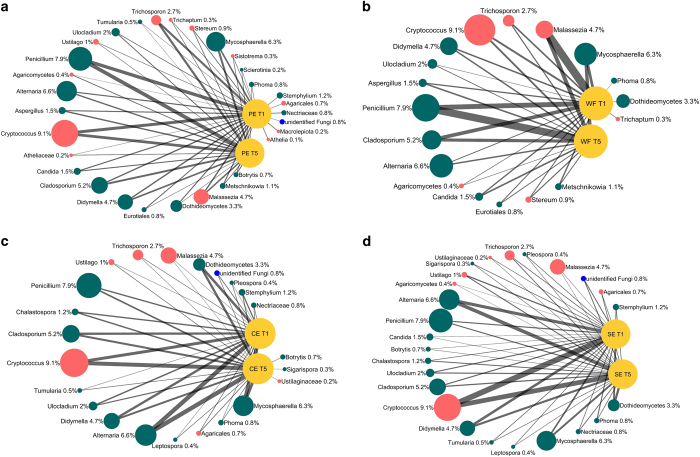
Network figures comparing fungal populations soon after fruit purchasing (T1) and after two weeks of storage (T5) in the calyx end (CE), stem end (SE), wounded flesh (WF), and peel (PE) of apple fruits. Networks were constructed regardless of practice (using data from organic and conventional apples) and considering only significantly different taxa (*P*<0.01) with a cumulative RA in organic and conventional apples ⩾0.1%. Investigated samples were represented by large yellow nodes. Detected taxa were represented by green (Ascomycota), red (Basidiomycota), and blue (Unidentified fungi) nodes. The size of nodes is proportional to the cumulative RA of each taxon in all investigated samples. Similarly, the percentage values reported along taxa represent the cumulative RA of each taxon in all investigated samples. On the contrary, the width of line connecting samples and fungal taxa is proportional to the amount of each specific taxon in each specific sample.

Overall, time only had a minor impact on the composition and RA of fungal communities present on apple fruit, regardless of whether they were grown organically or conventionally. No significant difference in alpha diversity was observed, although a significant difference in beta diversity was revealed in CE samples of organic and conventionally grown apples collected at T1 versus T5 (Table 2). Regardless of the statistical significance, some interesting changes in the fungal population at T5, relative to T1, were observed. Six and three taxa were exclusively detected at T1 in PE and WF samples, respectively (Figure 8). In contrast, three taxa were detected only at T5 in CE samples (Figure 8), and the RA of *Penicillium* in WF samples was higher at T5 than it was at T1 (indicating that it had increased over time). An opposite trend was observed for *Malassezia*.

## Discussion

Results of the present study reveal the complexity of the composition of fungal communities associated with fruit tissues and how they can be modulated by many different factors including fruit morphology (location), management practices, wounding, and time. A major finding of the study was the detection of significantly different populations of fungi in the different locations (CE, SE, WF, and PE) of the apple, even though, they were collected from the same fruit. The differences in the spatial composition of fungal communities was highly significant in regard to both alpha and beta diversity, and were true in both organic and conventionally grown apples and at both assessment times (soon after fruit purchase and after two weeks of storage at room temperature). Several factors are likely to have contributed to the observed differences. Apple fruit can be divided in several parts based on morphological and biochemical characteristics that may have an effect on the fungal species inhabiting each microenvironment. Longitudinally apple fruits can be divided into at least three locations: the top of the fruit which includes the stem end (pedicel), the middle part (core) which includes the seeds, and the calyx end which includes the remains of the apple flower parts. Apple flowers are epigynous so the base of the flower parts are fused to form a hypanthium and the ovary is embedded in the hypanthium. Apple fruit can also be described as being composed of three layers, the exocarp (peel), the mesocarp (flesh), and the endocarp (core).

The findings of the current study provide significant new information on the fungal microbiota of harvested fruit tissues, some of which is surprising. Available data on the microbiota of aerial plant parts, and in particular the phyllosphere, may help in the interpretation of the obtained results, although most previous investigations have focused on bacteria rather than fungi. Different plant parts (leaves, flowers, fruits, or stems) have been reported to influence the structure of the resident microbial communities.^28,29^ Leaf surfaces are characterized by different microenvironments brought about by the presence of a diverse, and heterogeneous set of morphological structures (hairs, waxes, thick cuticles and so on) resulting in an uneven distribution of epiphytic bacteria.^30–33^ For example, the morphological and structural characteristics of the adaxial and abaxial leaf surface has a strong impact on the resident microorganisms that are present.^34^ The effect of these parameters on the composition of the resident microflora has been attributed to an uneven distribution of nutrients, water, and humidity; to variations in temperature and exposure to UV light; and to the differential presence of potential gateways for penetration into the plant endosphere.^35^ Similarly, the topography and structural composition of the fruit surface, especially of the SE and CE, are likely to play an important role in sheltering microorganisms and protecting them from UV light and other adverse environmental conditions. In this regard, both the SE and CE are sunken areas in which water and nutrients can accumulate and supply microorganisms to a greater extent than other portions of the fruit. The CE also includes the remains of flower parts (sepals, stamens and the style) that represent a preferential substrate for many necrotrophic fungi and therefore can facilitate their colonization and survival, as well as the establishment of latent infections.^36,37^ Theoretically, one would predict that the equatorial zone of the fruit is the least favorable location for fungal establishment and proliferation since it is more exposed to UV light and its topography does not favor the accumulation of water and/or nutrients. This premise, however, does not seem to be supported by our data since the number of OTUs and other alpha diversity metrics did not reveal a lower diversity in PE samples. Importantly, however, the investigated surface area was much higher for PE samples and comprised mostly exocarp (peel) tissues. In contrast, the other investigated locations comprised both external and internal tissues (CE and SE) or only internal (mesocarp) tissues (WF).

Although caution should be used in extrapolating the results of the present study too far, significant differences in the composition of the fungal microbiota and the RA of different members of the microbiota were observed in organic and conventionally grown apples. These differences were consistent in all four of the investigated fruit parts. Interestingly, the differences observed between organic and conventionally grown apples were mainly in low abundant fungi and included both known plant pathogens such as *Botrytis* and *Phoma* but also fungi that represent potential antagonists, such as *Dioszegia*. Several taxa were exclusively detected in samples obtained from organic apples, suggesting that the chemicals commonly utilized in conventional agriculture may have an impact on non-target organisms. Glenn *et al.* ^37^ also found that apple leaves obtained from trees where an organic pest management system was used exhibited a larger unique phyllosphere microflora composition than leaves from trees utilizing conventional pest management practices, although the microbial composition was generally stable with high evenness.^38^ A lower overall genetic diversity in the phyllosphere, as a consequence of chemical applications, has been generally reported.^39–41^ It is important to note, however, that factors not accounted for in the present study, such as potential differences in the handling and processing of the two different fruit types (certified organic versus non-organic), may have also had a major role in the results obtained in the present study. More detailed, controlled studies would be needed to resolve this question. The general influence of the chemicals used in conventional management systems, such as synthetic fertilizers and disease and insect sprays, have been shown to have a direct impact on the fruit microflora. In a recent study, the suspension of chemical treatments for a single month was enough to modulate the fungal populations present on strawberry plants, although differences were more evident on leaves and flowers than fruits.^42^ Determination of the exact impact of the various inputs used in different management systems on the fungal microbiota will require more detailed studies.

In regards to the assessment of the impact of time on the fungal microbiota, little differences were observed between the fungal microbiota of apples soon after purchase (T1) and after 2 weeks at room temperature (T5). This result was consistent in samples obtained from both organic and conventionally grown apples. Significant differences in beta diversity indices were only observed in CE samples. The absence of significant differences due to time was surprising, particularly for the wounded flesh samples since conspicuous colonization of wounds by necrotrophic fungi would have been expected, especially since fruits were maintained at room temperature for 2 weeks. Although some differences, mainly related to the increased relative abundance of *Penicillium* spp. in wounds after 2 weeks, were observed, the changes were not enough to statistically support the presence of different populations. In agreement with the amplicon metagenomic analyses, none of the wounded apples developed evident rots during storage (data not shown).

Among the fungal taxa detected in this study, some were universally detected and exhibited no significant differences in their RA in different fruit parts (CE, SE, WF, and PE), management practices (organic versus conventional), or assessment time (T1 and T5). This was the case for important fungal genera such as *Mycosphaerella*, *Cladosporium*, and *Aureobasidium*. The abundant and uniform presence of these genera was not surprising considering that they are considered ubiquitous fungi. *Cryptococcus* was one of the most abundant genera observed and was especially prevalent in SE samples. This genus is comprised of species that form biofilms and melanin-like pigments that facilitate survival and reduce the sensitivity of fungal cells to heat, cold, and UV light.^43^ The abundant presence of *Cryptococcus* may be relevant considering that species in this genus have been used as biocontrol agents against apple post-harvest pathogens, including blue mold caused by *Penicillium expansum*,^44^ and gray mold caused by *Botrytis cinerea*.^45,46^ Interestingly, *Cryptococcus* species were abundantly detected in both organic and conventionally grown apples suggesting that this genus has a high tolerance to commonly used chemical treatments. This may be useful information for the development of integrated control strategies. Other potentially relevant antagonists detected in the present study included *Metschnikowia* and *Wickerhamomyces*, which were found almost exclusively in peel and wound samples. *Metschnikowia* species have been proposed as effective biocontrol agents against postharvest rots^47^ and play an important role in must fermentation.^48^ Species of *Wickerhamomyces* are known for their general antifungal activity and are considered safe, non-pathogenic organisms that can be used to control harmful yeasts and bacteria in the food industry.^49^ The ability of these microorganisms to live both outside and within fruit tissues may represent an advantage as a biocontrol agent since plant tissues provide shelter and nutrients and thus allowing microorganisms to develop under less competitive conditions and potentially shield interior plant tissues from pathogens.^3,50^

Species of *Penicillium* were also detected with a high RA and were the most abundant fungi in PE and WF samples with a RA of 9.90% and 15.90%, respectively. A phylogenetic analysis indicated that the most abundant *Penicillium*-related OTUs were associated with *P. expansum* and related species, including *P. digitatum* and *P. italicum* that cannot be differentiated based solely on their ITS2 sequences. Considering the role of *P. expansum* as a major apple pathogen, it is likely that most detected species belong to this species. Recent studies have demonstrated, however, that apart from *P. expansum*, several other *Penicillium* species, including *P. digitatum* and *P. italicum* can cause rots in artificially inoculated pome fruits.^51,52^ These infections can develop, however, only in over-ripe, senescing apples. Results indicated that the RA of *Penicillium* spp. increased in wounds over a two-week period indicating that wounded apple tissues are actively colonized by these pathogens, although the increase in the number of sequences detected for this genus was not enough to cause the development of visible rots.

Analyzed apple SE and CE samples had a higher RA of *Alternaria* compared to PE and WF samples. The abundant presence of *Alternaria* species in these samples is not surprising considering that these species are the major causal agents of core rot in apple fruit (i.e., a rot initiating in the endocarp and spreads into mesocarp tissues^53^). *Alternaria* colonizes senescing flower parts during and shortly after bloom and moves, presumably through the tube formed by the fused flower parts (calycine tube), into the receptacle or core of the fruit.^37,54^ Early studies on the etiology of *Alternaria* core rot of apple fruit reported *Alternaria alternata* as the causal agent of the disease, however, more recent studies have demonstrated the involvement of several *Alternaria* species, including those detected and phylogenetically identified (*A. alternata, A. tenuissima, A. infectoria*, and *A. arborescens*) in the present study.^55–57^ Interestingly, other non-identified fungi belonging to the same order as *Alternaria* (*Pleosporales*) were abundantly detected (22 and 64%) in CE and SE samples, respectively, and had a very low RA or were absent in PE and WF samples. Genera related to *Alternaria,* such as *Pleospora* and *Stemphylium,* were also much more abundant in core samples (CE and SE). BLAST searches showed a high similarity of the detected sequences to the species, *P. herbarum*, *S. vesicarium,* and *S. sarciniforme*, plant pathogens responsible for leaf and fruit spot in several crops, including apple.^58^ Another related genus (*Ulocladium* spp.) was also detected. Importantly, species in the order *Pleosporales*, and in particular *Alternaria* spp., are known to produce mycotoxins. Therefore, their presence in apples, even as latent infections, may pose a health risk, especially in processed apple products.^56^

Another fungal genus that was found to be highly prevalent in apple core samples, especially CE samples, was *Acremonium* spp. This genus comprises many species known as endophytes, saprobes in air or soil, or even as plant, animal, and human pathogens.^59–61^ One species, *A. sclerotigenum* was recently reported to cause *Acremonium* brown spot on bagged apple fruit in China.^62,63^ The phylogenetic analysis conducted in the present study supported the presence of *A. sclerotigenum* in our samples but it was less abundant than the other detected taxa such as *A. alternatum* and *A. egyptiacum.* Some species of *Acremonium* have been proposed to serve as biocontrol agents against different plant pathogens, including *Venturia inaequali*s, the causal agent of apple scab.^64–66^ *Acremonium* spp. is also considered an important endophyte and has been associated with resistance to insects but also associated with toxicosis in livestock.^67,68^ Considering the abundant detection of this genus in apples, further investigations are warranted to determine its role as a pathogen or antagonist in apple.

In contrast to *Acremonium,* the genus *Didymella* (teleomorph *Phoma s. str*.) was found mainly in SE samples, although it was present to some degree at all of the sampled fruit locations. *Phoma s. str*. fungi are associated with many land plants ^69–71^ and in agreement with their abundant detection in the SE samples, may cause stalk end rot in apple.^72^ Most of the species putatively detected and phylogenetically confirmed in the present study, including *D. glomerata, D. pomorum*, and *Phoma macrostoma,* are known to cause leaf spot diseases in apple.^73^

A conspicuous number of other fungal taxa, belonging to the genera *Malassezia, Candida*, and *Trichosporon*, were not expected to be found on or in apples but were identified in the present study. The genus *Malassezia* is comprised of yeasts found in the cutaneous microflora of humans and other warm-blooded animals, and can be involved in disorders including dandruff and seborrheic dermatitis.^74^ Among the species phylogenetically identified in the present study, *M. restricta* is known to cause keratitis in humans and *M. globosa* and *M. sympodialis* are the causal agents of pityriasis versicolor. Sequences of *Candida* spp. were mainly identified as *C. quercitrusa, C. sojae, C. parapsilosis*, and *C. oleophila.* Except for *C. oleophila*, which is a common inhabitant of fruit tissues and has been demonstrated to be an effective postharvest biocontrol agent,^75^ the other *Candida* species are frequently isolated from healthy humans but can also cause symptomatic infections of mucosal membranes.^76^ The genus *Trichosporon* was mainly represented by the species, *T. moniliiforme* and *T. porosum* and by an unidentified species related to *T. jirovecii* and *T. cutaneum*. The first two species have been previously isolated from soil and decomposing leaves,^77,78^ while the second two species are known to be infectious to humans.^79^ In general, *Trichosporon* species are typical causal agents of cutaneous infections and are involved in systemic, localized, or disseminated mycoses in humans.^80^ In addition, a number of *Trichosporon* species are being reported to be involved in causing mortality in immunocompromised patients.^81^ The identification and potential impact of the mentioned species as potential human pathogens should be interpreted with great caution due to the small sample size used in this study, the general lack of knowledge about the natural microbiota of produce, and the difficulty in confirming species identification in metagenomic studies based solely on ITS sequence data. The results have no implications regarding the impact of organic versus conventional management practices on the presence on specific taxa, especially those associated with human health.

The most obvious speculation about the origin of human fungal pathogens on apple fruit is their contamination from human skin during harvesting, or other points of contact in the distribution chain (packing, shipping, and store display), although, the abundant presence of these fungi in flesh tissues that had no direct contact with humans does not support the contamination hypothesis. The role of plants as alternative hosts for human and animal pathogens has been reported but most of the available data has focused on the interactions between plants and human pathogenic bacteria.^82^ It is generally accepted that human pathogenic bacteria can adapt to plant hosts despite the fact that plants present a different physiology, immunity, native microflora, physical barriers, mobility, and temperature than would be present in human hosts. Although similar information for fungi is very limited, a similar adaptation may be possible for human pathogenic fungi. Indeed, *Malassezia restricta* has been isolated as an endophyte from *Populus deltoides*,^83^* Spiranthes spiralis*,^84^ and *Solanum tuberosum*.^85^ It was also found on orchid roots^86^ and in soil nematodes.^87^

The existence of unknown plant endophytes that are very closely related to human pathogens but not in themselves pathogenic to humans is another possible explanation for the detected taxa. Indeed, the existence of related fungal species with very similar or even identical ITS sequences but with completely different pathogenic characteristics has been reported. For example, strains of the species *Ustilago*, a typical plant pathogen, can infect humans.^88,89^ The phylogenetic position of the genus *Malassezia* indicates a close relationship to plant pathogens, implying a possible ancestral shift from plant to animal host preference.^90^ As previously mentioned, it is also important to highlight that the species identification designated in the current study must be viewed with caution since it is only based on the analysis of the ITS2 sequence and because of the complex taxonomy of most fungal families.^91^ The combination of standard bioinformatic analyses with specific BLAST and phylogenetic analyses, combined with the accurate selection and validation of reference sequences, however, should have enabled the identification of the detected taxa with a high level of accuracy.^15,27^

In conclusion, results of the present study have provided a deeper insight into the fungal communities associated with apple fruit. Some of the detected taxa were already known as apple-associated fungi but many other taxa were detected for the first time or represented completely unknown taxa. The presence of unidentified fungi, even at the phylum level, indicates that the fungal diversity of apple fruit, as well as in other plant species and organs, is far from being completely characterized. The comprehensive picture of the fungal diversity provided in the present study may serve as a foundation for future investigations focusing on specific groups of fungi. Unfortunately, the limited genetic variation within the analyzed barcode gene (ITS2 region) did not enable the precise identification of several of the detected taxa which limited discussion about their importance and role. In this context, further analyses of the apple fungal microbiota with other more variable barcode genes seem to be worthwhile. More detailed information on the spatial and compositional variation of the fungal microbiota of apple as they are impacted by location, management practice, wounding, and storage time, are needed but will require more controlled studies. The present study lays the foundation for these future studies.

## Figures and Tables

**Table 1:** Summary of analyses and alfa diversity results of metagenomic surveys conducted with conventional and organic apples, soon after fruit purchasing (T1) and after 2 weeks of storage (T5), and with different fruit parts including stem end (SE), calyx end (CE), wounded flesh (WF), and peel (PE)

	*Total reads*	*Total OTUs*	*OTUs 700*^a^	*Shannon*	*Chao1*
*Conventional*					
T1					
SE	194159	364	34.6	3.166448	75.83333
CE	125725	298	22.9	1.934228	50.07833
WF	10372	78	37.8	2.510214	43.90085
PE	129369	332	95.3	4.784009	139.6858
T5					
SE	84698	192	29.4	2.682104	63.12667
CE	116059	289	15.6	1.591507	21.25000
WF	53464	138	42.4	2.740644	65.18015
PE	80009	183	77	4.568145	101.1823
					
*Organic*					
T1					
SE	111066	275	40.5	2.993430	66.99952
CE	79089	162	23.5	1.498342	33.40952
WF	30538	135	67.3	4.957833	76.63969
PE	65419	143	56	4.051488	75.27202
T5					
SE	96374	205	46.5	3.757823	78.33488
CE	401759	441	24.4	2.724301	39.44333
WF	160605	346	24.6	2.437313	36.17345
PE	97221	237	51.4	3.337052	77.45083

a OTUs determined at an even depth of 700 sequences.

**Table 2:** Statistical significance of differences (*P*-values) among fungal population detected in investigated apple samples

	*Conventional versus organic*^a^	*T1 versus T5*^b^	*Sample location (conventional)*^a^			*Sample location (organic)*^a^		
			*SE*	*WF*	*PE*	*SE*	*WF*	*PE*
CE	0.001	0.042	0.002	0.004	0.002	0.001	0.001	0.001
SE	0.003	0.057		0.001	0.001		0.001	0.001
WF	0.020	0.062			0.006			0.003
PE	0.002	0.157						

The comparison regarded conventional and organic apples, fruits soon after their purchasing (T1) and after two weeks (T5) and different apple parts including calyx end (CE), stem end (SE), wounded flesh (WF), and peel (PE). This latter comparison was performed for both conventional and organic apples. Significance was determined according to the nonparametric test Permanova and the beta diversity metric Bray Curtis.

a Samples from Conventional and organic apples were analyzed together.

b Samples from both assessment times (T1 and T5) were analyzed together.

**Table 3:** Comparison of alpha diversity according to Shannon index in different apple parts including stem end (SE), calyx end (CE), wounded flesh (WF), and peel (PE)

	*WF*	*PE*	*CE*	*SE*
WF	2.683643	3.055868	4.026681	0.073815
	1.07277	0.0096	0.003	0.934
PE	—	3.855272	10.11071	5.188563
		0.682745	0.002	0.003
CE	—	—	1.369323	−6.62464
			0.569226	0.006
SE	—	—	—	2.661566
				2.561939

Analyses were performed regardless of time (soon after fruit purchasing and after two weeks of storage) and regarless of regardless of the practices (conventional and organic apples). Gray boxes contain mean values (top line) and standard deviation (bottom line) determined for each apple part. White boxes contains *t*-test results (top line) and false discovery rate *P*-value (bottom line).
