# Supplementary material for: Spatial and compositional variation in the fungal communities of organic and conventionally grown apple fruit at the consumer point-of-purchase
Source: Hortic Res. 2016 Oct 5;3:16047–. doi: 10.1038/hortres.2016.47 (PMC5051542; doi:10.1038/hortres.2016.47)
Supplement: Supplementary Material [file hortres201647-s1.doc]

Supplemental Material 1. Phylogenetic identification of detected sequence types (STs). Trees were built using unique sequences representative of STs of the most relevant fungal genera detected in the present study and validated reference sequences of each fungal genus.

B

C

D

G

A

F

E
